# Home-Based Exercise to Improve Functional Outcomes in Veterans With a Recently Healed Diabetic Foot Ulcer: Protocol for a Pilot Randomized Controlled Trial

**DOI:** 10.2196/71237

**Published:** 2025-09-23

**Authors:** Gwen L Robinson, Jake Drumheller, Alison D Lydecker, Bailey Rammling, Elizabeth A Dennis, Odessa Addison, Steven J Prior, Brock A Beamer, John D Sorkin, H David Gottlieb, Kiana Trent, Mary-Claire Roghmann

**Affiliations:** 1 VA Maryland Health Care System Baltimore, MD United States; 2 School of Medicine University of Maryland, Baltimore Baltimore, MD United States; 3 Baltimore GRECC VA Maryland Health Care System Baltimore, MD United States; 4 Department of Kinesiology University of Maryland, College Park College Park United States

**Keywords:** rehabilitation, home-based exercise, exercise, diabetic foot ulcer recurrence, clinical trial, veteran, diabetic foot ulcer, foot ulcer, diabetes

## Abstract

**Background:**

Foot ulcers are a common complication of diabetes, often resulting from peripheral neuropathy and inadvertent trauma. Poor healing is exacerbated by peripheral arterial disease and poor glycemic control. Off-loading, a key treatment, leads to prolonged immobility. Patients rarely regain baseline mobility. Mobility is crucial to improve glycemia, promote vascular health, and improve immobility as it leads to nursing home admissions. There is limited research on exercise during ulcer remission.

**Objective:**

This pilot study will assess the feasibility and acceptability of a home-based exercise regimen aimed at safely increasing mobility and function, focusing on improving lower extremity strength, tissue perfusion, and glycemic control.

**Methods:**

Veterans aged ≥50 years with a recently healed diabetic plantar foot ulcer receiving care in the US Department of Veterans Affairs (VA) Maryland Health Care System and enrolled in a remote temperature-sensing mat program will be eligible. Potential participants will be identified via administrative codes used for the Prevention of Amputation in Veterans Everywhere directive, as well as using the VA’s Podimetrics SmartMat dashboard. In this pilot study, 25 veterans will be randomized (in a 3:1 ratio) to a 12-week home-based exercise regimen or standard of care. Participants will undergo tests for gait speed, knee extension strength, cutaneous perfusion, and community mobility. The intervention group will participate in internet-based videoconference exercise classes twice a week led by the study team and home cycling 3 times a week. The control group will receive standard-of-care guidance. Outcome measures will include feasibility; acceptability; and changes in gait speed, physical activity levels, and strength.

**Results:**

This study was funded on July 1, 2024, with data collection planned from October 1, 2024, to March 31, 2026. The protocol was approved by the University of Maryland Institutional Review Board on May 13, 2024, and by the Baltimore VA Research and Development Committee on June 13, 2024. As of June 12, 2025, 12 participants have been enrolled in the study, and 6 (50%) participants have been randomized. Recruitment is expected to continue through December 2025.

**Conclusions:**

This project has potential for clinical rehabilitation translation. If it is found to be feasible and acceptable, the exercise intervention will be tested in a future multisite randomized clinical trial to assess its impact on mobility, cardiovascular events, and ulcer recurrence.

**Trial Registration:**

ClinicalTrials.gov NCT06312579; https://clinicaltrials.gov/ct2/show/NCT06312579

**International Registered Report Identifier (IRRID):**

DERR1-10.2196/71237

## Introduction

### Background

A total of 38.4 million Americans live with diabetes, with another 1.2 million people diagnosed each year [1]. For people living with diabetes, foot ulcers are a common and feared complication, with 1 in 20 people developing a foot ulcer at least once during their life [2]. The pathway to ulceration is well established and involves a combination of multiple comorbid conditions [3]. An example of this combination includes diabetes with peripheral neuropathy and inadvertent trauma to the foot leading to a wound. The resulting wound fails to heal and becomes a chronic ulcer, in part because of poor tissue perfusion and poor glycemic control.

People with a diabetic foot ulcer (DFU) have a higher 5-year mortality than closely matched people with diabetes but no ulcer―1 in 4 people with a DFU will die within 5 years, largely due to cardiovascular causes [4,5]. The reasons for this accelerated mortality are not well defined but likely involve dysmobility and inflammation. Following the development of an ulcer, part of treatment is to take pressure off the ulcer, known as off-loading. Off-loading attempts to remove the mechanical stress from the tissue associated with the ulcer. Healing can take months, leading to a prolonged loss of mobility [6,7]. While off-loading is one of the most important interventions, often seen as critical to the healing of a plantar foot ulcer, there is little done to restore mobility following off-loading. The risk of muscle atrophy and mobility loss during ulcer treatment is substantial due to disuse related to off-loading. It is likely that the combination of dysmobility, deconditioning from inactivity, and increased inflammation from the ulcer contribute to the high level of adverse cardiovascular outcomes following remission from a DFU.

People who have healed a DFU are in remission. They are at high risk of ulcer recurrence because the underlying factors that led to their ulcer typically have not changed [8]. Once in remission, the current standard of care is to advise a slow increase in ambulation so as not to promote ulcer recurrence; however, patients in recovery from a DFU frequently do not return to their preulcer level of mobility [9-12]. Currently, there is a dearth of studies that examine the effects of exercise during the remission phase. We currently know of only 1 small randomized study that found a decrease in ulcer recurrence and improvement in peripheral neuropathy and walking speed [13]. This provides a clear opportunity for further research.

### Objectives

Our intervention was designed based on previous successful telehealth interventions used in the US Department of Veterans Affairs (VA) Gerofit program and our other VA research project, as well as evidence in the literature that similar, multimodal exercise protocols improve gait speed in older adults [14]. It meets the American College of Sports Medicine guidelines to improve mobility and strength while maintaining the safety of those at high risk of falls and recurring foot ulcers. There is growing evidence that people with diabetes and peripheral neuropathy can safely maintain a recommended level of activity [15-17]. These results have led to changes in the American Diabetes Association and the International Working Group on the Diabetic Foot guidelines to allow for weight-bearing exercise in those with peripheral neuropathy without severe foot deformity or open lesion [18,19]. A 3:1 randomization (intervention to control) will allow us to assess the feasibility and acceptability of the intervention with greater precision while maintaining a standard-of-care control arm. A comparison of mobility between the intervention and control arms will give us an estimate of effect size for future sample size calculations.

Given previous studies and our experience with Gerofit, we hypothesize that a home-based exercise regimen will increase mobility and function without increasing DFU recurrence by improving lower extremity strength, lower extremity tissue perfusion, and glycemic control.

## Methods

### Study Design and Overview

**Figure 1:**
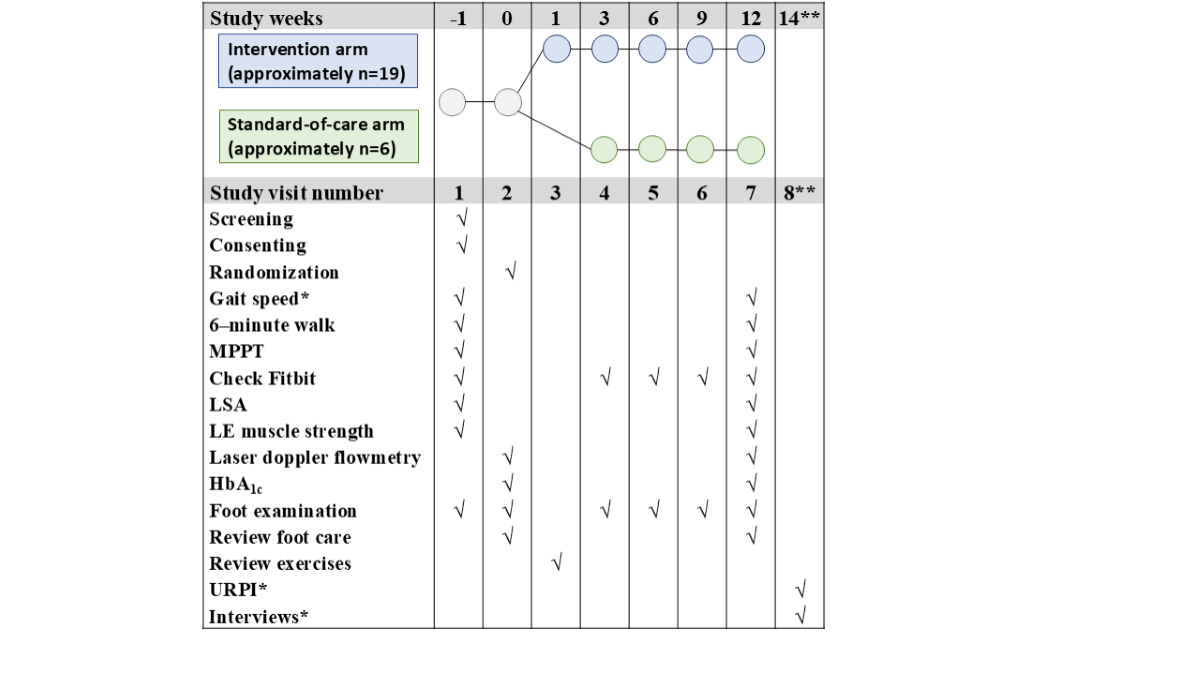
Clinical trial study design. HbA1c: glycated hemoglobin; LE: lower extremity; LSA: Life-Space Assessment; MPPT: Modified Physical Performance Test; URPI: Usage Rating Profile–Intervention; *Primary outcome; **Phone call only, intervention group only.

This study is a randomized, single-blind (the investigator who assessed the outcome was blinded to exercise assignment), controlled clinical trial of exercise versus standard of care. This study was funded by the VA as a Small Project in Rehabilitation Research as a pilot study to inform sample size calculations for future larger clinical trials. Therefore, we will recruit 25 veterans with a recently healed DFU who will be randomized at a ratio of 3 intervention participants to 1 control participant to reach a final sample size of 20, anticipating a 20% loss to follow-up. There will be a total of 7 in-person study visits and 1 phone call for those in the intervention group and 6 in-person study visits for those in the standard-of-care group, as shown in Figure 1. The study timeline is shown in Table 1. The investigator assessing the functional outcomes will only perform visits 1, 2, and 7 (Figure 1). They will not run any of the exercise sessions or follow-up study visits (visits 3-6). During study meetings, they will also be recused from the meeting when it is time to talk about randomization and follow-up visits. Those in the intervention group will participate in a 12-week home-based exercise program led by an exercise physiologist. The exercise group will exercise 5 days a week, including 3 days a week of recumbent cycling and 2 days a week of remote group strengthening exercises using resistance bands. Every 3 weeks, participants will attend an in-person study visit to ensure that they are exercising safely, modify or advance their exercise regimen by increasing their band resistance, reinforce proper foot care, and have the integrity of their feet examined by a trained staff member. Participants in the standard-of-care group will also attend an in-person session every 3 weeks to examine their feet and reinforce proper foot care. At baseline or randomization and after 12 weeks, mobility and function for all participants will be measured as outlined in Table 2 and detailed in Figure 1. The analysis will compare the change in outcome measures between the week 12 visit (visit 7) and the randomization visit (visit 2) within individuals between the intervention and control arms. For those in the intervention group, the feasibility and acceptability of the intervention will be assessed using the Usage Rating Profile–Intervention (URPI) [20,21] and semistructured interviews 2 weeks after completion of the intervention.

**Table 1:** Study timeline with number of participants enrolled and randomized.

Task	October 2024-March 2025	April 2025-September 2025	October 2025-March 2026	April 2026-September 2026
Project start-up―team training and stakeholder engagement	✓			
Recruitment	✓	✓	✓	
Participants enrolled, n	8	4	—^a^	—
Participants randomized, n	5	1	—	—
Study procedures―each participant is in the study for 12 weeks	✓	✓	✓	✓
Data collection and management	✓	✓	✓	✓
Statistical analysis and dissemination of results				✓

^a^Not available.

**Table 2:** Baseline and end point assessments^a^.

Assessment	Description
Gait speed (primary outcome)	Usual gait speed will be calculated using standard protocols based on a 10-minute walk distance. We will provide individuals with a 14-minute path to provide room for a “flying start” to determine gait speed without the confounding factor of acceleration and deceleration [22,23].
Mobility function―6-minute walk distance and MPPT^b^ (secondary outcome)	Mobility function will be assessed using the MPPT (a 9-item standardized test) [24] and 6-minute walk distance. Through standard procedures, participants will be asked to walk as far as possible in 6 min along a 100-foot course. Total distance, heart rate, and number of breaks taken will be recorded.
Physical activity―steps per day (secondary outcome)	Physical activity will be assessed as average steps per d measured using a Fitbit Inspire 3 (Google), which will be provided to the participants and worn for the duration of their participation in the study.
Community mobility (secondary outcome)	Community mobility will be assessed using the Life-Space Assessment [25,26].
Strength (secondary outcome)	Knee extension and plantar flexion strength will be assessed using a handheld dynamometer, as we have previously described [27]. Three repetitions will be performed on each leg, with rest between each repetition.
Perfusion (secondary outcome)	Cutaneous perfusion will be assessed on the dorsal and plantar surfaces of the foot using laser Doppler flowmetry. Briefly, the laser Doppler probe will be positioned on either the dorsal or plantar surface of the foot between the first and second metatarsals. Baseline measurements of perfusion are obtained, and the probe is gently heated to 44 °C, causing dilation to assess maximal perfusion in the area of interest [28].
Glycemic control (secondary outcome)	Blood samples will be obtained through venipuncture for HbA_1c_^c^ measurement and future storage.

^a^All end points will be measured at both the randomization visit and the week 12 visit.

^b^MPPT: Modified Physical Performance Test.

^c^HbA_1c_: glycated hemoglobin.

This study protocol follows the SPIRIT (Standard Protocol Items: Recommendations for Interventional Trials) guidelines [29]. The study will follow the CONSORT (Consolidated Standards of Reporting Trials) guidelines and reporting requirements. Any deviations from the protocol, breaches of confidentiality, and reportable adverse events will be reported to the VA Office of Research and Development Institutional Review Board (IRB) and Data Safety Monitoring Board (DSMB). In addition, the DSMB will review study-related materials biannually and review collected data to ensure data integrity, security, and control for quality assurance. This study is registered at ClinicalTrials.gov (NCT06312579).

### Participants and Inclusion and Exclusion Criteria

Community-dwelling veterans aged ≥50 years (N=25) with diabetes and a recently (within the previous 3-27 months) healed plantar foot ulcer who are enrolled in the VA Maryland Health Care System (VAMHCS) and currently using or willing to use a remote monitoring temperature-sensing mat (Podimetrics SmartMat program) are eligible for this study. Additional inclusion criteria are having 2 feet (can have healed minor amputations of the fore and midfoot), having appropriately fitted therapeutic footwear for exercise, being ambulatory without use of a walker, and being able to provide written informed consent. Individuals will be excluded if they are unable to perform the exercise interventions; have a foot surgery planned in the following 4 months; are currently participating in an exercise or rehabilitation program; are currently diagnosed with a plantar foot ulcer or preulcer; or meet any other criteria that, in the investigators’ opinion, would compromise the ability of a participant to take part safely in the intervention.

### Recruitment and Screening

All study participants will be recruited from the VAMHCS patient population. They will be identified via administrative codes used for the Prevention of Amputation in Veterans Everywhere (PAVE) [30] directive, as well as using the VA’s Podimetrics SmartMat dashboard. PAVE is a VA directive that requires VA medical centers to track veterans by their risk of future lower extremity amputation. The Podimetrics SmartMat dashboard provides a list of veterans who are currently enrolled and participating in the SmartMat program, as well as those who are eligible. We will use the PAVE administrative codes to identify potential participants through their history of diabetes, recent history of DFU, and last amputation. We will cross check those from the PAVE database with a recently healed foot ulcer with those listed on the Podimetrics SmartMat dashboard. We will use other recruitment modalities, including but not limited to individual letters, posters in clinics, electronic bulletin boards, and My HealtheVet (VA patient health care portal). Veterans who respond to the recruitment efforts and agree to screening will undergo an initial telephone screen to assess eligibility and interest in the study. Individuals who meet the eligibility criteria will provide written informed consent to study coordinators, provide demographic information, and undergo a medical history and physical examination with a licensed medical provider. This visit will include an assessment of their current footwear, how it fits, and any skin breakdown on the feet to ensure that participants can safely take part in the exercise intervention. Eligible participants will then be scheduled for a baseline assessment and randomization.

### Baseline Assessment and Final Measures

An assessment battery will be administered before randomization and after 12 weeks of participation in each study group (exercise or standard of care). All baseline assessments and testing will be conducted at the Baltimore VA Medical Center over 1 to 2 visits during a 1-week period and will be collected using standardized protocols and by trained research team members. These assessments are described in detail in Table 2 and include the following: gait speed over 10 meters, 6-minute walk distance, the Modified Physical Performance Test, steps per day and community mobility, strength, perfusion of the foot using laser Doppler flowmetry, and glycemic control as assessed through glycated hemoglobin.

### Randomization

Participants will be randomized after baseline testing during visit 2. Performing the screening and randomization visits on different days helps ensure that the participant is willing and able to return for future study visits, which increases the chances of randomized participants adhering to the intervention. Study participants will be assigned to 1 of 2 arms (exercise and standard of care) using blocked randomization with a 3:1 allocation ratio. Randomization allocation, generated by the study statistician, will be concealed in sequentially numbered opaque sealed envelopes. The envelopes will contain a card with study arm allocation and a piece of carbon paper. These envelopes will be stored in a locked filing cabinet and will be used in sequential order individually at the time of randomization. Participant study ID, date and time of randomization, and the signature of the person performing the randomization will be written on the outside of the envelope and transferred via the carbon paper to the card with study arm allocation before the envelope is opened.

### Exercise Intervention

#### Overview

Consistent with current physical activity recommendations for older adults [31], participants will be prescribed 5 days per week of exercise, with home-based seated cycling exercise performed 3 days a week using a study-provided DeskCycle 2 under-desk bicycle pedal exerciser and strength and balance exercises performed 2 days a week in an internet-based videoconference group exercise class. All exercises are designed to minimize weight bearing and reduce shearing forces to the skin of the feet. The initial exercise session will be conducted in the VAMHCS exercise facility under the direction of trained study staff to ensure that participants are familiar with the equipment and receive proper instruction on the exercise prescription. If it appears that the participant needs more assistance, a follow-up in-person session will be scheduled. The remaining exercise sessions will be home based, where the cycling exercise will be performed independently and the strength and balance exercises will be performed in an internet-based videoconference group class. Participants will also return for 1 in-person session every 3 weeks to check participants’ feet and adjust the exercise prescription. We will use SMS text messaging or phone calls to increase adherence to the exercise regimen. The intervention will be paused if the participant is felt to have a safety concern (eg, skin breakdown, such as a blister or redness) pending clinical evaluation to ensure safety.

#### Cycling Exercise

Participants will be provided with a DeskCycle 2 under-desk bicycle pedal exerciser in their home for seated cycling (3 days per week). Initially, participants will be asked to accumulate 20 minutes of cycling at 50 to 60 revolutions per minute per day at a “fairly light” intensity using the Borg rating of perceived exertion (RPE) scale (RPE=1-2 on a scale of 1-10). Participants may not be able to complete 20 consecutive minutes of cycling, so they will be permitted to accumulate 20 minutes through ≥2 bouts of 5 to 10 minutes. During weeks 2 to 3, participants will progress the duration of cycling, with a goal of 30 consecutive minutes of cycling per day. From weeks 4 to 12, participants will continue cycling for 30 minutes per day but will increase the intensity to an RPE of 7 to 8 (“somewhat hard” but still a relatively low overall exercise intensity) using resistance adjustments on the DeskCycle 2 under-desk bicycle pedal with guidance from the study team. Participants will be provided with a Fitbit (Google) to use throughout the study, and data from the Fitbit will be synchronized to anonymized accounts for objective tracking of time, workload, and overall adherence to the protocol.

#### Strength and Balance Exercises

The strength and balance exercises will be performed during an internet-based videoconference group exercise session (2 days per week) led by members of our study team. These sessions will begin with a brief check-in followed by a seated warm-up. Participants will then be led through 10 different exercises, all of which can be performed using a standard kitchen or dining chair or similar (leg press, knee extension, plantar flexion, squat, chest press, chest pull, row, lateral raise, biceps curl, and triceps curl). Participants will then be led through sit-to-stand exercises and standing balance exercises (ie, tandem stance, single-limb stance, and narrow base of support) with a chair nearby for support and safety. Finally, participants will perform a seated cooldown. The entire internet-based videoconference session will take between 45 and 60 minutes. Progress will be assessed, and new resistance bands will be provided at the triweekly in-person visits to adjust the exercise prescription. Attendance, participation, and Fitbit heart rate data will be used to confirm adherence to the protocol. These internet-based videoconference sessions also allow for a visual inspection of the feet if any concerns arise.

### Standard of Care

The participants who are randomized to the standard-of-care group will be provided with guidance on the current standard of care, namely, that they should slowly increase ambulation with appropriately fitted footwear. We will examine their feet at each study visit every 3 weeks to ensure safety. Should any evidence of ulceration be noted, appropriate referrals will be facilitated to podiatry and orthotics. We will review appropriate foot care with each participant at each study visit. If appropriate, all randomized participants from both groups will be offered ongoing participation in a VA clinical exercise program at the completion of the trial. Participants in the standard-of-care arm will receive a Fitbit as well to compare step count to that of the intervention group but will not be given any guidance for exercise.

### Retention

Participant study visit scheduling will be made in conjunction with participant outpatient visits at the VAMHCS if feasible. Participants will be either texted or called before the study visit as a reminder. Study participants in the intervention arm will also be texted weekly via the Annie app as a reminder to engage in the in-home exercise intervention. The Annie app is a new VA-approved texting program that provides a full audit trail of all text communications with participating veterans. Participants will also be encouraged to call if they have foot-related issues. In the event that there is a foot-related issue, we will facilitate a visit to podiatry at the VAMHCS. Information on the Annie app can be found on this website [32]. Those in the intervention arm will also check in with a study team member twice a week during the internet-based videoconference exercises.

Participants will be asked to come in for a final visit to conduct final outcome assessments if they wish to withdraw from the study after randomization. Participants in the exercise arm will also be scheduled for the semistructured interview and URPI survey. If they are unable to participate in a final visit or are unwilling or unable to undergo the semistructured interview or URPI, no further data collection will be conducted.

### Assessment Measures

#### Primary Outcomes

##### Feasibility and Adherence

A flowchart will be prepared to identify and summarize recruitment and retention (ie, total number screened and excluded and timing and frequency of dropouts). Acceptable recruitment will be defined as 90% of the targeted enrollment within 15 months of initiation. Acceptable retention will be assessed as 80% participation in the exercise intervention and study visits. Adherence to the exercise will be assessed using a combination of three methods: (1) written logs of home-based exercise (resistance, time, and RPE) returned to study staff every 3 weeks when participants report for in-person visits; (2) attendance during the internet-based videoconference group strength exercise sessions; and (3) anonymized Fitbit accounts established for each participant and used to download heart rate, cycling, and step count data to verify completion of exercise during the logged times.

##### Acceptability

Acceptability and feasibility of the intervention for the participants will be measured using the URPI [20] and through a semistructured interview. The URPI feasibility (eg, “I would be able to allocate my time to do this intervention”) and acceptability (eg, “The intervention is a good way to improve my walking”) subscales, which were developed and validated with an ethnically diverse sample, use a 6-point Likert scale from 1 (strongly disagree) to 6 (strongly agree). Higher scores indicate greater feasibility and acceptability. A score of “agree,” or >3, on the URPI will be used to quantify that the intervention is acceptable and feasible. We will use an inductive thematic approach for the semistructured interviews, which will provide qualitative feedback regarding participants’ perceptions of the program, including feasibility of participating and areas for improvement that will be used to tailor a future intervention. We plan to use a convenience sample drawn from participants in the intervention arm of the trial as we are seeking their perspectives on the feasibility and acceptability of the intervention. Participants will be interviewed by a research team member who has experience working with older veterans. Interviews will be conducted either by telephone or in person at the final study visit depending on the participant’s preference and overall convenience. Questions will include the following: (1) “Why did you decide to participate in the program?” (2) “What are some things about the program that made it easy for you to complete the exercise intervention?” (3) “What are some things about the program that made it harder for you to complete the exercise sessions?” (4) “Do you think you will continue with the exercise program after this study has ended? Why or why not?” (5) “What do you need to help you succeed with maintaining a consistent exercise routine in the long term?” (6) “How can we help make it easier for other Veterans to participate in an exercise program like this?” (7) “Is there anything else you’d like to share with us today related to the exercise program?” Interviews will be recorded using a HIPAA (Health Insurance Portability and Accountability Act)-compliant program and transcribed, and a template will be developed to gather key points from the interview guide. A codebook based on the interview guide will be used to find emergent themes and patterns. To understand factors influencing the future implementation and sustainment of our home exercise intervention, we will also code transcripts to identify implementation barriers and facilitators.

#### Secondary Outcomes

Other data to be collected will include demographics, current and past foot conditions, peripheral neuropathy and its treatment, location of recently healed ulcer, usual foot self-care, type of footwear, complications and control of diabetes, peripheral arterial disease and its treatment from the medical record, and participant interview and examination.

### Data Analysis

The primary objectives assessed in this study include recruitment, retention, adherence to the exercise intervention, acceptability of the intervention, and change in gait speed. The analyses for all primary objectives will focus solely on the randomized participants except for recruitment, which will also include enrolled but not randomized participants. We will use the mean and SD of the within-person change (pretest-posttest value) in gait speed. A flowchart (ie, CONSORT flowchart) will be prepared to identify and summarize recruitment and retention (ie, total number screened, numbers excluded, and timing and frequency of dropouts). Retention will be assessed through the count of those who drop out. Please see the section primary outcomes for how feasibility and adherence will be analyzed. This statistical analysis plan and protocol will be posted on ClinicalTrials.gov.

The acceptability of the intervention will be gauged using the URPI and qualitative interviews among those randomized to the intervention either when they finish the intervention or after study withdrawal. We will calculate averages. For qualitative data, we will use inductive thematic analysis, where we will collect these data and articulate patterns within them, and these patterns will collectively explain what occurs within the data. We will code transcripts to identify themes (eg, reported experiences) using qualitative analysis software, review patterns in core themes, determine the degree of overlap, and develop a network diagram of interrelationships between themes. Themes will be used to describe correlates of acceptability of the exercise intervention and identify factors that are likely to influence participation in the program. We intend to use multiple coders, and we will analyze intercoder reliability using 25% of the sample, aiming for a Cohen κ of >0.75 [33,34]. In addition to examining the coder agreement, we will use member checking [35]. We will share preliminary analyses with selected participants and ask them to confirm whether the data reflect their perspectives and what they discussed in their interviews. Finally, we will be mindful that the researchers’ experiences may be different from those of the study participants. We will bring awareness of the potential influence on data collection, data analysis, and how we perceive the data, as well as how the participants perceive the researchers conducting the study, when conducting the qualitative analyses [36,37].

The estimate of the effect of the intervention on gait speed and other secondary outcomes will be determined through the change in outcome measures between intervention end (ie, week 12 study visit) and intervention start (randomization). We will use the mean and SD of the within-person change (pretest-posttest value) in gait speed in a sample size calculation for a future randomized clinical trial. We will conduct analyses with and without adjustment for adherence to the intervention by incorporating the proportion of exercise sessions completed (ie, percentage of adherence) as a factor in the analyses.

All data will be assessed for missingness and impossible or out-of-range values using basic statistical procedures such as univariate statistics. Missing data will be imputed using multiple imputation, which provides unbiased estimates if the data are missing completely at random or at random. There will be no interim analysis.

### Participant Safety and Minimizing Potential Risk

The risks from participating in the research program include minor discomfort, but these are transient. Participants will undergo procedures that involve a mild to moderate degree of risk, including questionnaires, exercise training, and mobility assessments. The interviews and questionnaires in this study are time-consuming but of minimal risk. Completion of physical activity is associated with the risk of cardiovascular complications such as chest pain, myocardial infarction, or sudden death and complications related to stress and strains of muscles, twisted ankles, or falls. The American Heart Association consensus statement on exercise standards [38,39] estimates that the acute risk of sudden cardiac arrest during exercise training in participants with known cardiac disease is approximately 1 event per 58,000 hours of aerobic exercise. The risk of exercise training is greater at higher exercise intensities. This risk is offset by prescreening participants with medical evaluations before exercise and starting them out at a low intensity. A research team member (exercise physiologist or other medical professional) certified in American Heart Association Basic Life Support will be present during all internet-based videoconference exercise sessions. We will follow standard protocols for our facility regarding the use of internet-based videoconference exercise to ensure safety. In addition, there is a minimal risk of falling during the walking and mobility tests. A standby aid is always present, and a gait belt is used to increase safety when necessary. Ample rest periods are provided to limit fatigue during testing.

At each study visit, participants will be asked whether they had any adverse events since the last study visit. Adverse events that occur during study visits or internet-based videoconference exercise classes will also be captured. These adverse events will be systematically recorded and reviewed by the study principal investigator. Adverse events will be classified based on whether they are study related, a serious adverse event, and unanticipated or unexpected. Adverse events will be collated and then reviewed biannually by the Veterans Integrated Service Network (VISN) 5 Geriatric Research, Education, and Clinical Center DSMB and reported to the IRB as necessary. The board’s composition and standard operating procedures have been documented and approved by the IRB at the University of Maryland, Baltimore. Its primary focus is safety, particularly monitoring expected and unexpected adverse events directly attributable to study participation (ie, a fall during an exercise class).

### Data Management

Participants will be assigned a unique study ID number that will be kept in a password-protected database stored on a VA server with a level and scope of security that equals or exceeds that established by the HIPAA Security Rule. Data collected during the study that are not captured electronically will be entered and stored in the VA REDCap (Research Electronic Data Capture; Vanderbilt University) server. Participants’ charts will be held in a locked room inside a locked cabinet at the Baltimore VAMHCS. Data will be inspected for completeness, out-of-range values, missingness, and internal consistency (when relevant). Access to the individual participant study ID numbers or personal identifiers will be limited to research team members who need to know to perform their study role.

Study data will be kept in accordance with the VA Records Control Schedule 10-1. Storage and transfer of any personally identifiable information or protected health information must be conducted in accordance with applicable VA and Veterans Health Administration policies and directives; state and federal regulations; and applicable statutes, including HIPAA.

### Protocol Modifications

The study team, comprising the principal investigator, coinvestigator, study coordinators, data manager, and study manager, have weekly meetings. Any protocol modifications are communicated in these meetings or via email. There are also monthly meetings with the entire team to track progress and check in.

### Ethical Considerations

This protocol was approved by the University of Maryland IRB (the IRB of record for the Baltimore VAMHCS) on May 13, 2024, as a minimal risk study, and by the Baltimore VA Research and Development Committee on June 13, 2024. In addition, written informed consent will be obtained from all participants before participating in the study. The participants’ privacy will be kept confidential and they will be informed of their right to withdraw their consent at any time. All participants will be reimbursed with US $50 for study visits 1 to 7 for their time and effort and US $25 for the semistructured interview.

### Dissemination

The results of this study will be made available to health care professionals and the public through the National Library of Medicine PubMed Central website within 1 year after the date of publication. In addition, study findings will also be presented at scientific meetings. Study investigators will be responsible for the writing of all publications and will not use the services of professional writers.

## Results

This study was funded on July 1, 2024, with a projected data collection period from October 1, 2024, to March 31, 2026. As of June 12, 2025, there are 12 participants who have been enrolled in the study, and 6 (50%) participants have been randomized.

## Discussion

### Expected Findings

This pilot study will assess the feasibility and acceptability of a home-based exercise regimen aimed at safely increasing mobility and function, focusing on improving lower extremity strength, tissue perfusion, and glycemic control. The feasibility of, adherence to, and acceptability of the intervention will be assessed. While we anticipate that people in the intervention group will have increased mobility and function, a decrease in major adverse cardiovascular events, and a decrease in long-term ulcer recurrence, this study is designed to be a pilot study. As such, any measures of physiological change with exercise are not expected to demonstrate statistical significance and are being collected solely to provide effect size estimates that will be used to develop sample size estimates for a larger intervention efficacy trial. This research is important because those who have DFUs rarely return to their baseline level of mobility, much less a recommended level of mobility [9-12]. The period after ulcer healing is a critical time to intervene and could improve mobility and restore function. It could also have a high impact on a population with a clear history of health disparities through the ability to exercise from home. This intervention has clear potential to be scalable as a telehealth group intervention.

The study outlined in this paper has several notable strengths that enhance its potential impact. First, the use of the SmartMat for safety monitoring is a significant strength as it allows for daily remote tracking of foot temperature, which can help in early detection of potential issues such as ulcer recurrence. This proactive approach to safety ensures that participants can engage in the exercise regimen with reduced risk. Second, the detailed tracking of participation through written logs, internet-based videoconference session attendance, and Fitbit data provides a comprehensive view of adherence and engagement. In addition, the use of semistructured interviews and the URPI to assess the barriers and acceptability of the intervention offers valuable qualitative insights. These methods allow for a deeper understanding of participants’ experiences and challenges, which can inform future improvements to the program. Finally, the home-based exercise regimen, combined with objective monitoring of adherence, supports participants in maintaining their mobility and function in a convenient and accessible manner. This approach not only promotes sustained engagement but also has the potential to be scalable and adaptable to other populations, enhancing its overall applicability and impact.

This study has several limitations. One limitation is the lack of diversity within the participant population, which predominantly consists of male veterans. This sex imbalance may limit the generalizability of the findings to the broader population of individuals with DFUs, particularly women, who may have different responses to the intervention. In addition, the study’s focus on veterans from a specific health care system may not capture the variability in outcomes that could occur in a broader, nonveteran population. Another limitation is the potential preference for in-person exercise programs among this patient population. While the home-based exercise regimen offers convenience and accessibility, some participants may prefer the structure, social interaction, and direct supervision provided by in-person programs. This preference could affect adherence and engagement levels, potentially affecting the study’s outcomes. Addressing these limitations in future research could enhance the applicability and effectiveness of the intervention across a more diverse and representative sample.

### Future Directions and Conclusions

Our long-term goal is to improve clinical outcomes for adults with a DFU by improving their treatment and rehabilitation. This pilot study is expected to provide preliminary evidence for a larger-scale, randomized clinical trial that will examine the exercise intervention tested in this pilot study and result in improved mobility and function in those with a recently healed ulcer while also minimizing ulcer recurrence. On the basis of the findings of this project, these future clinical studies may address adaptations to the exercise intervention, changes in cardiovascular risk following exercise, and long-term ulcer recurrence with increased supervised activity. Future trials will be needed to validate these findings before broad-based acceptance and dissemination across VAs can occur. Thus, the proposed research will not only provide information for the veterans participating in this study but will also lay a foundation for future research targeting veterans and other people with recently healed foot ulcers.

## Abbreviations

CONSORT: Consolidated Standards of Reporting Trials
DFU: diabetic foot ulcer
DSMB: Data Safety Monitoring Board
HIPAA: Health Insurance Portability and Accountability Act
IRB: Institutional Review Board
PAVE: Prevention of Amputation in Veterans Everywhere
REDCap: Research Electronic Data Capture
RPE: rating of perceived exertion
SPIRIT: Standard Protocol Items: Recommendations for Interventional Trials
URPI: Usage Rating Profile–Intervention
VA: United States Department of Veterans AffairsVAMHCS: United States Department of Veterans Affairs Maryland Health Care System

## Data Availability

The final datasets underlying all publications resulting from the proposed research will be shared outside the US Department of Veterans Affairs. The datasets generated or analyzed during this study are available from the corresponding author on reasonable request.
